# Characterization of a hypothetical protein YVRE from Bacillus subtilis indicates its key role as glucono-lactonase in pentose phosphate pathway and glucose metabolism

**DOI:** 10.6026/97320630013430

**Published:** 2017-12-31

**Authors:** S.V. Reshma, Nitish Sathyanarayanan, H.G. Nagendra

**Affiliations:** 1 Department of Biotechnology, PES University, Bangalore; 2 Department of Biotechnology, Sir M Visvesvaraya Institute of Technology, Hunasemaranahalli, Bangalore 562157; 3 Present Address: National Centre for Biological Sciences, Tata Institute for Fundamental Research, GKVK Campus, Bellary Road, Bangalore 65

**Keywords:** Hypothetical protein, Bacillus subtilis, SMP-30/Gluconolactonase/Regucalcin, Gluconolactonase, pentose phosphate pathway, glucose metabolism

## Abstract

Hypothetical proteins are functionally uncharacterized proteins with assigned function using sequence annotation tools. Almost half of the coding regions of several genomes are hypothetical proteins. Therefore, it is of our interest to characterize a hypothetical protein YVRE from the model system Bacillus subtilis using known data. YVRE is assigned the function as a glucono-lactonase using prediction and phylogenetic analysis. A molecular dynamics simulated homology model of YVRE (with calcium) using human senescence marker protein 30 /SMP30 (PDB ID: 3G4E) as template is reported for functional inference. It is observed that the protein possesses bivalent metal binding domain. Molecular docking studies with the substrate glucono-δ-lactone show YVRE binding with the substrate. This data was further validated using cloning and sub-cloning in pUC57 and pET22b+ respectively, followed by expression and purification using nickel affinity chromatography. The activity of YVRE using the substrate glucono-δ-lactone was calculated. The results show the function of YVRE as a gluconolactonase, with higher preference to zinc than calcium or magnesium. Thus, YVRE is shown to play key role in three metabolic pathways namely, pentose phosphate pathway, ascorbate and aldarate metabolism, and caprolactam degradation.

## Background

Genomes contain the information and operating capabilities that determine the structure and function across biological organization. Exploration of these systems offers a comprehensive way of understanding the modes by which biological entities operate in nature. Substantial portion (around 30-40%) of any sequenced genome, encode for hypothetical proteins and efforts are on to characterize this special class of molecules. Elucidation of structure and function of hypothetical proteins is imperative to understand the biological system in toto. Understanding the function of a protein include knowledge of biochemical activity, biological process and evolutionary aspects [1]. Hence, the need for approaches to reveal the functions of all hypothetical genes in a sequenced genome are significantly emphasized [2].

Conserved hypothetical proteins pose a challenge not only to functional genomics, but also to general biology. Often, a general prediction of the function of hypothetical protein can be made based on a conserved sequence motif, subtle sequence similarity to a previously characterized protein or the presence of diagnostic structural features [3]. Structural genomics initiatives also facilitate a thorough investigation toward assigning functions to vital genes and enable delineating the functions of hypothetical proteins, which might play key roles in cellular functions. However, integrating the techniques related to computational biology, comparative genomics, mutational analysis and curation might help in identifying the most intriguing genes in every genome. Many conserved hypothetical genes have been confidently predicted to be ATPases, GTPases, methyltransferases, DNA/RNA binding proteins etc. [4]. Similarly, application of computational tools and prediction methodologies have offered equally valuable clues in recognizing the functional aspects of proteins under study, as well. These in silico results could be used as indicators for establishment of actual function of proteins via experimentation and analysis. In a similar endeavour, our group has assigned the function of a hypothetical protein VNG0128C from Halobacterium NRC-1 as UDP-galactose 4-epimerase involved in galactose metabolism [5].

Gluconolactonase was first reported in yeasts in 1955 by Brodie and Lipmann [6] and since then has been reported in bacteria, fungi, plants and animals. It catalyses the conversion of Dglucono- 1,5 lactone to D-gluconate Expression of Gluconolactonase in Psedomonas aeruginosa was demonstrated to cleave D-glucono-δ-lactone and found to be important for its fitness and growth [7]. It belongs to Senescence Marker Protein 30 (SMP-30)/Gluconolactonase/Regucalcin superfamily. Expression of SMP-30, the animal gluconolactonase, was shown to decrease androgen-independently with aging [8].

## Methodology

Computational studies: A multi-step insilico characterization of the hypothetical protein YVRE (GI ID: 16080373), was performed to decipher the plausible function of the uncharacterized protein Domain association was performed using well known tools such as InterProScan [9] and CDD (conserved domain database) [10], to understand the inherent signatures present within the primary sequence of the protein. Jackhmmer [11] was used for retrieving the sequence from Swiss-Prot (characterized) database (with an e value of 0.001). The hits collected after 3 iterations were analysed for their domain composition using hmmscan [11], to remove false positives. A total of 25 true homologs were obtained. Phylogenetic analysis was carried out using MEGA v5.1 [12]. 10 iterations of multiple sequence alignment were performed using MUSCLE available within MEGA v5.1. The tree was built using Maximum likelihood method with 100 bootstrap replications. The output was visualised with FigTree.

BLAST search was performed against the PDB database to find a suitable template for homology modelling of the target sequence YVRE. While ClustalW [13] was used to generate the alignment between template and query, Modeller [14] was used for generating a 3D model. Though 100 models were generated, the model containing the best Discrete Optimized Protein Energy (DOPE) score was chosen for further analysis. PyMol was used to visualize the modelled structures. LeadIT tool as part of the FlexX [15] was used for docking purposes. Ca2+ ion, one of the possible divalent metal ion cofactors was docked manually by superposing the model with the template structure. Further, to identify the Pharmacophore, the modelled structure was used as a query to search for possible PDB structures having a bound substrate or substrate analogue. This search was performed using 3D-BLAST. The substrate analogue, D-Xylitol which is cocrystallized in 4GNA (mouse SMP30/GNL-xylitol complex [16]), was docked onto the model to understand the residue level conservation at the catalytic binding pocket between the template and the model. The substrate, D-glucono-1, 5-lactone was also docked onto both template and model, to elucidate the pharmacophoric patterns. The substrate analogue D-xylitol was first re-docked to the template to account for any deviations within the algorithm.

To further understand the residues critical for binding of divalent metal ion, the model and Ca2+ (manually docked structure) was subjected to molecular dynamics simulation using GROMACS 5.0 [17]. The simulation was performed using Optimized Potentials for Liquid Simulations (OPLS) force field, by solvating the protein in a cubic box. Upon neutralization of the system with addition of required ions, the protein was energy minimized using steepest descent algorithm. Further to this, equilibration of the system was performed using NVT and NPT ensembles. 100 ps simulation of each of the steps were undertaken, and the total simulation run was performed for 6 ns.

### In vitro studies

The experimental characterizations involved cloning and expression of the protein, and enzymatic assays. Bacillus subtilis (ATCC 6051/JCM 11081) culture was obtained from Microbial Type Culture Collection-IMTECH, Chandigarh, India. The strain was cultured at pH 7.0 and 300C on nutrient agar medium containing 5g of sodium chloride, 3g of beef extract, 5g of peptone and 2% agar. Upon isolation of genomic DNA using standard protocols [18] the gene was amplified using manually designed sequence specific primers. PCR amplified products were digested with HindIII and BamHI restriction enzymes, and cloned into pUC57 and subsequently sub cloned into pET 22b+ expression vector containing C terminal poly-histidine tag. The integrity of the clone was verified by sequencing.

The clone was further transformed into chemically competent E. coli BL21 cells compatible to the expression vector. Single colonies were grown at 370C in 5 ml of LB media containing 100 μg/ml ampicillin until the value of the A600 reached 0.8-1.0. 1mL aliquot of each culture was induced by 0.5mM isopropyl-1-thio-β-Dgalactopyranoside (IPTG) for expression at different time periods (at an interval of one hour each for 6 hours) at 370C at 200 rpm. Induction was monitored using SDS-PAGE and, fusion protein was purified using nickel affinity column following manufacturer's instructions. Purification was done under native conditions. The pellet was re-suspended in 10mL native lysis buffer and incubated on ice for 30 minutes. The lysate was centrifuged at 14000 rpm for 30 minutes at 40C to remove the debris. The supernatant was applied to the column and eluted using imidazole buffer. Fractions corresponding to maximum peak at 280nm were pooled and further analyzed using electrophoresis on 10% SDS-polyacrylamide gel. Protein estimation was done by Lowry's method [19].

Isoelectric focusing (IEF) was calculated by using the broad range ampholytes forming a pH gradient. 10% poly-acrylamide gel was prepared by adding 30% acrylamide-bisacrylamide, 30μL of broad range (pH 3-9) ampholytes mixture, 10μL TEMED and 50μL of the protein sample into tubes of the apparatus. Upon solidification, the tubes were fit into the apparatus and gel was run at 90C. Initial voltage was set to 200V and gel was run till the voltage reached zero. Staining of the gel was done uisng 0.1% Coomassie Brilliant Blue G 250 overnight and de-stained in distilled water.

Gluconolactonase activity was determined by using D-glucono-δ- lactone (Sigma) as substrate by colorimetric assay described by Hucho and Wallenfels [20]. Measuring the decrease in absorbance of para nitrophenol, a pH indicator, monitored conversion of Dglucono- δ-lactone. Opening of lactone ring causes the formation of H+ ions which acidifies the medium leading to a decrease in absorbance of para nitrophenol [7]. The decrease in pH is indicated by reduction in yellow colour at 405nm. 2mL of the reaction mixture contained 10mM PIPES buffer (pH 6.4), 10mM Gluconolactone, 1mM para nitrophenol (PNP), 1mM ZnCl2 and 100μL of the recombinant protein. Absorbance at 405nm was measured at 240C. Similarly, a blank was maintained without adding the recombinant protein in order to track spontaneous hydrolysis of para nitrophenol. Test for effect of divalent metal ions calcium and magnesium were also performed with 1mM of CaCl2 or MgCl2 in place of ZnCl2 in the aforementioned reaction mixture.

For calculation of specific activity of gluconolactonase, the method of Petek et al. [21] for α -galactosidase was followed. Hydrolysis of PNP per minute was calculated by using Beer- Lambert Law (εPNP = 18.5 mM-1.cm -1) and the equation: Units/ enzyme = ΔA405min Recombinant protein- ΔA405min Blank Volume of assay (Dilution factor) 618.5 (Volume of recombinant protein). Where, 6 are the Conversion factor for 6 minutes to 1 minute. Consequently, Units/mg for protein was calculated by using the concentration of recombinant protein (1.5 mg/ml). One unit of activity is defined as the amount of enzyme that converted 1 μmol of para-nitrophenolate to para-nitrophenol per minute at pH 6.4 and at 240C.

## Results & Discussion

**Figure 1:**
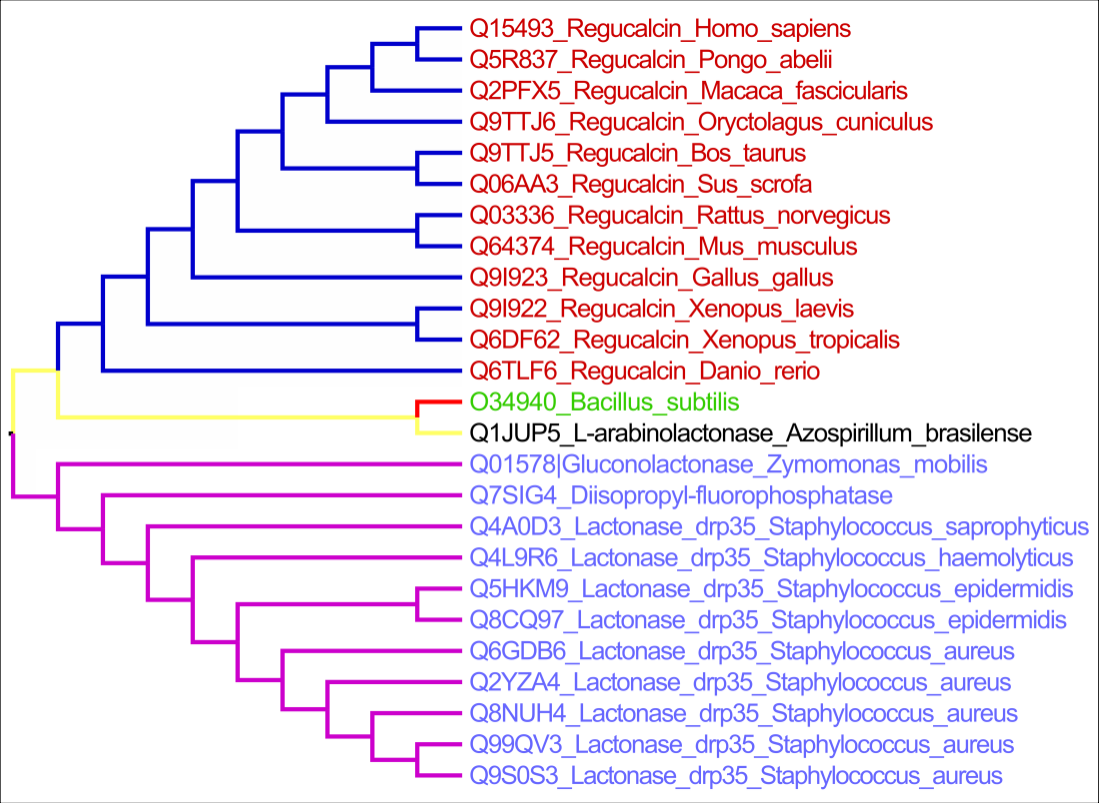
Phylogenetic tree of uniprot homologs along with query of interest from Bacillus subtilis.

The results of domain analysis indicated the presence of SMP30/Gluconolaconase domain. This family of proteins (PFam ID: PF08450.7) utilize divalent metal ions such as Ca2+ as a cofactor to convert D-glucono-1,5-lactone to D-gluconate. To further understand and predict the plausible function of the hypothetical protein, phylogenetic analysis was performed. The tree representation is provided in Figure 1. Interestingly, YVRE does not cluster either with the eukaryotic SMP/Regucalcin, which is involved in calcium homeostasis or with DRP35 (Drug response protein - 35 kDa), predominantly found in Staphylococcus, which proposes its plausible function as gluconolactonase.

**Figure 2:**
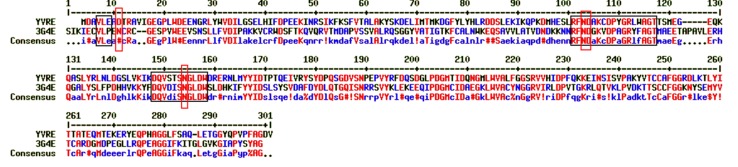
Alignment of template (PDB ID: 3G4E) and query YVRE.

To further understand the structure-function correlation, homology modelling was performed using PDB: 3G4E [22], that was obtained from the PDB search. This template (crystal structure of human senescence marker protein 30 /SMP30), shares an identity of 32.6% with the query sequence. The alignment of the query with the template that was used to derive the 3D model of the hypothetical protein sequence YVRE is depicted in Figure 2.

**Figure 3:**
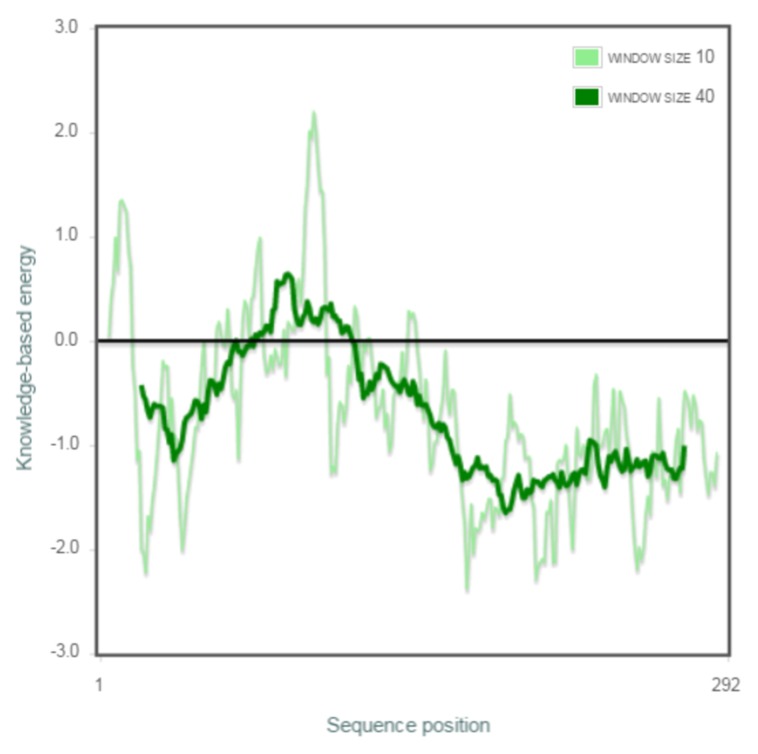
Residue-wise energy plot obtained from PROSA

The best model, chosen based on DOPE score was validated using several well-known methods. The RMSD for C-alpha atoms between the modelled structure and template was found to be 0.22 Å (for 98% of the residues superposed). The quality of the model was assessed with PROCHECK [23] which indicated that 97.2% of the residues were in allowed regions and only 2.8% noncritical residues were scattered in the disallowed regions of the Ramachandran map. PROSA [24] analysis was also performed where the model received a Z-score of -7.03. The residue wise energy plot is shown in Figure 3, and it depicts that there are no unstable segments in the protein [thick green line which is smoothened by calculating the average energy over each 40- residue fragment s (i, i+39)]. All these parameters suggest that the 3D model of YVRE is satisfactory.

**Figure 4:**
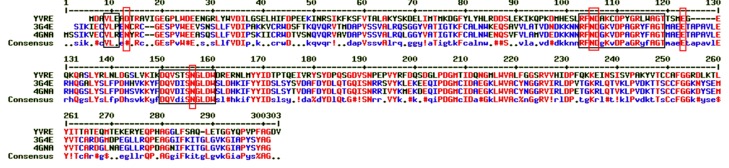
Alignment of query YVRE, templates (PDB ID: 3G4E and PDB ID: 4GNA). The Black box depicts conserved site of the substrate glucono-1, 5-lactone while the red box highlights the divalent ion cofactor binding site.

PDB: 3G4E, which was used for modelling YVRE, was the best template in terms of sequence identity and coverage but does not contain any bound ligand/substrate in the crystal structure. Hence, a 3D-Blast was performed using YVRE as a query to identify PDB: 4GNA with D-Xylitol, a substrate analogue bound in the crystal structure. 4GNA also belongs to the SMP-30 superfamily and the catalytic region is well conserved as shown in Figure 4. Also, PDB: 3G4E and PDB: 4GNA share a good structural similarity with RMSD of 0.95 A for all atom superposition.

The modelled protein was docked with the ligand glucono-1, 5- lactone and substrate analogue D-Xylitol. To appreciate the residue level interaction of the protein with its ligands, the information from 4GNA (which is a co-crystallized structure containing Ca2+ and D-Xylitol) was used to determine the residues around binding pockets. Further, the evolutionary conservation of these residues across a larger evolutionary space was determined using ConSurf [25], where standard algorithm parameters were employed.

**Figure 5:**
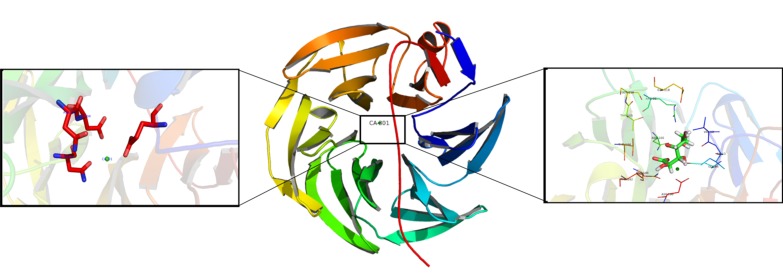
Structure of the model (top view) with bound Ca2+ and D-xylitol.

**Figure 6:**
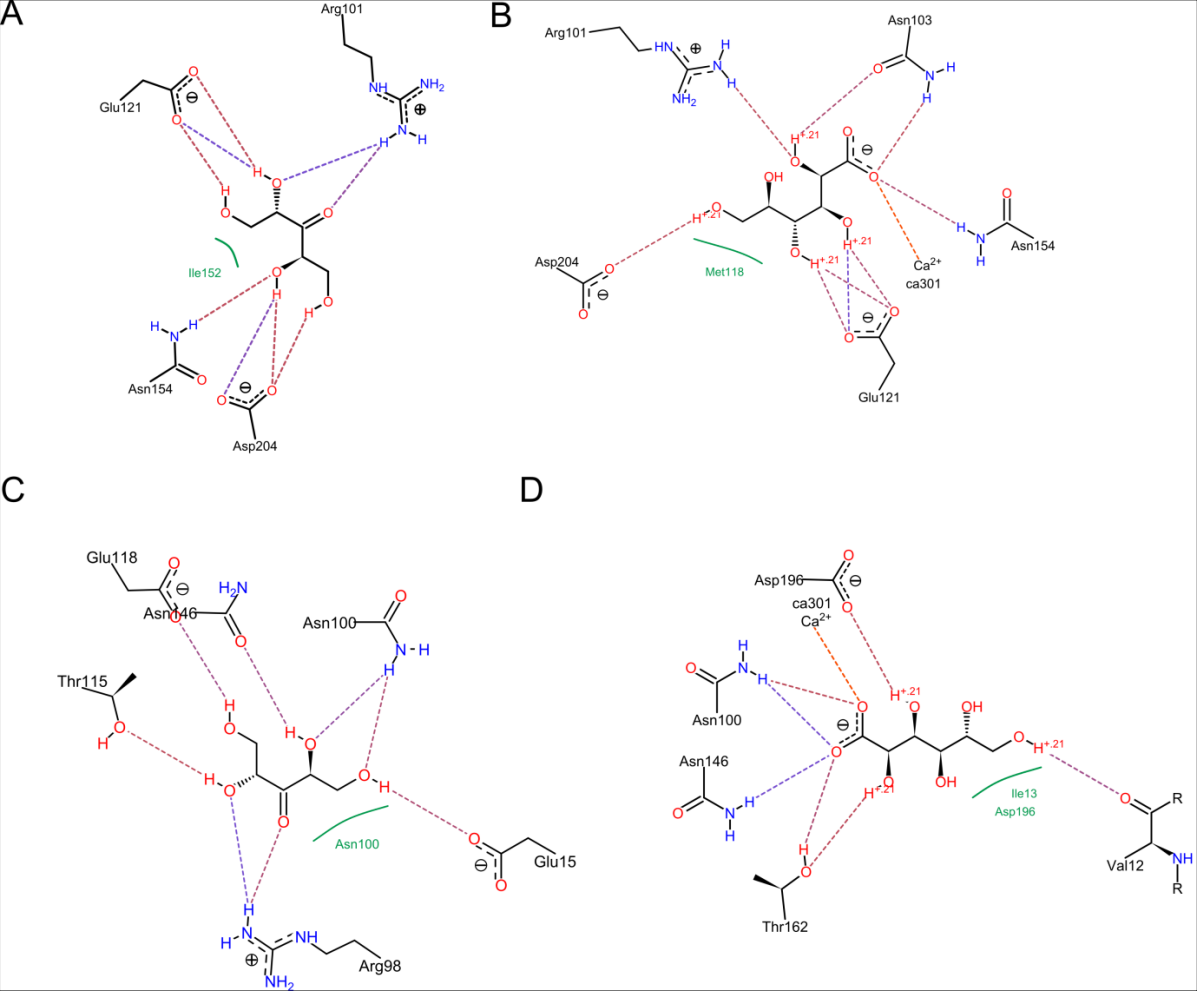
Ligplot of docked molecules with the protein model. A) D-Xylitol docked to 4GNA. B) D-glucono-1, 5-lactone docked to 4GNA. C) D-Xylitol docked to model. D) D-glucono-1, 5-lactone.

With this information, docking studies were performed using the standard parameters via the program FlexX to determine the nature of protein-ligand interaction for both the ligands. Figure 5 shows the cartoon representation of the model along with docked Ca2+ ion and substrate. Figure 6 depicts the 2D representation of the interactions with ligands, illustratively provides the schema of interactions with the ligands, in both the template structure and the modelled geometries. Though the substrate binds to the same regions, the RMS deviation of docked poses of glucono 1,5- lactone between the template (4GNA) and model is 2.4Å, while RMSD of docked poses for D-xylitol between template (4GNA) and model is 1.8Å. Table 1 provides a comparison of the residues around 5Å distance from the ligand. Residues in bold are evolutionarily highly conserved as obtained from ConSurf, and residues highlighted via the star-marks form hydrogen bond. Table 2 summarizes various statistical values (score and clash) and number of hydrogen bonds formed, as obtained during docking.

**Figure 7:**
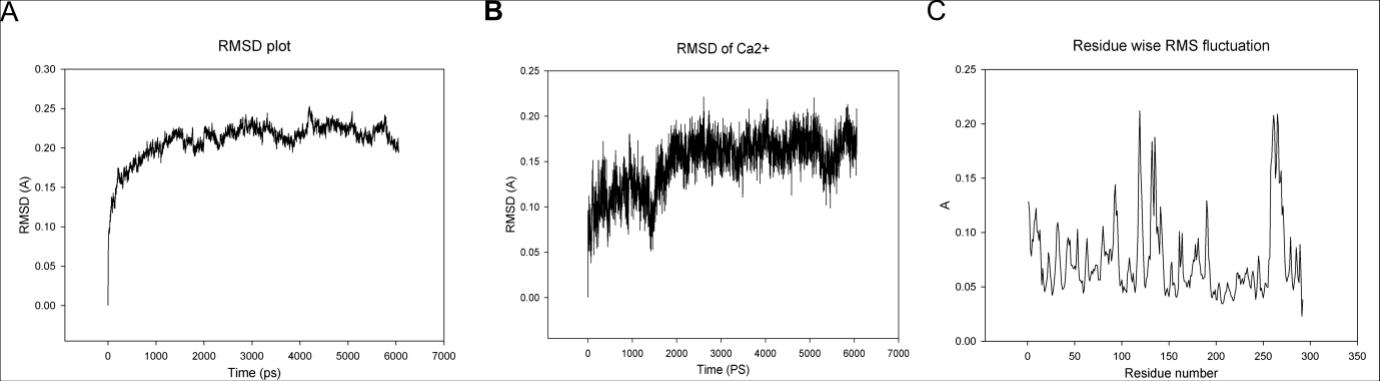
(A) RMSD plot of the protein; (B) Ca2+ RMSD plot; (C) RMSF plot for 6 ns simulation of the protein.

Furthermore to verify the interaction of Ca2+ to its binding residues E15, N100, D101 and N146, molecular dynamics simulation of 6ns was performed. The RMSD plot is depicted in Figure 7A. It is evident that the protein molecule does not show any large RMSD, and hence appears to be highly stable in solvent environment. Also RMSD plot of Ca2+ (Figure 7B) shows no large movement of the atom supporting the manual docking of Ca2+. The average B-factor of the Cα atoms of residues E15, N100, D101 and N146 determined through g_rmsf program have values 8.27, 13.62, 8.36, 5.09 respectively indicating that they are critical for binding of divalent metal ion. The RMSF plot (shown in Figure 7C), also clearly suggests no big residue level movement in any region of the protein, highlighting its stability during binding.

Thus, the phylogenetic anaylsis, homology modelling, molecular docking and MD studies clearly suggest binding of a divalent metal ion such as Ca2+ and glucono-1, 5-lactone to the hypothetical protein, and its plausible function as gluconolactonase.

### In vitro studies

**Figure 8:**
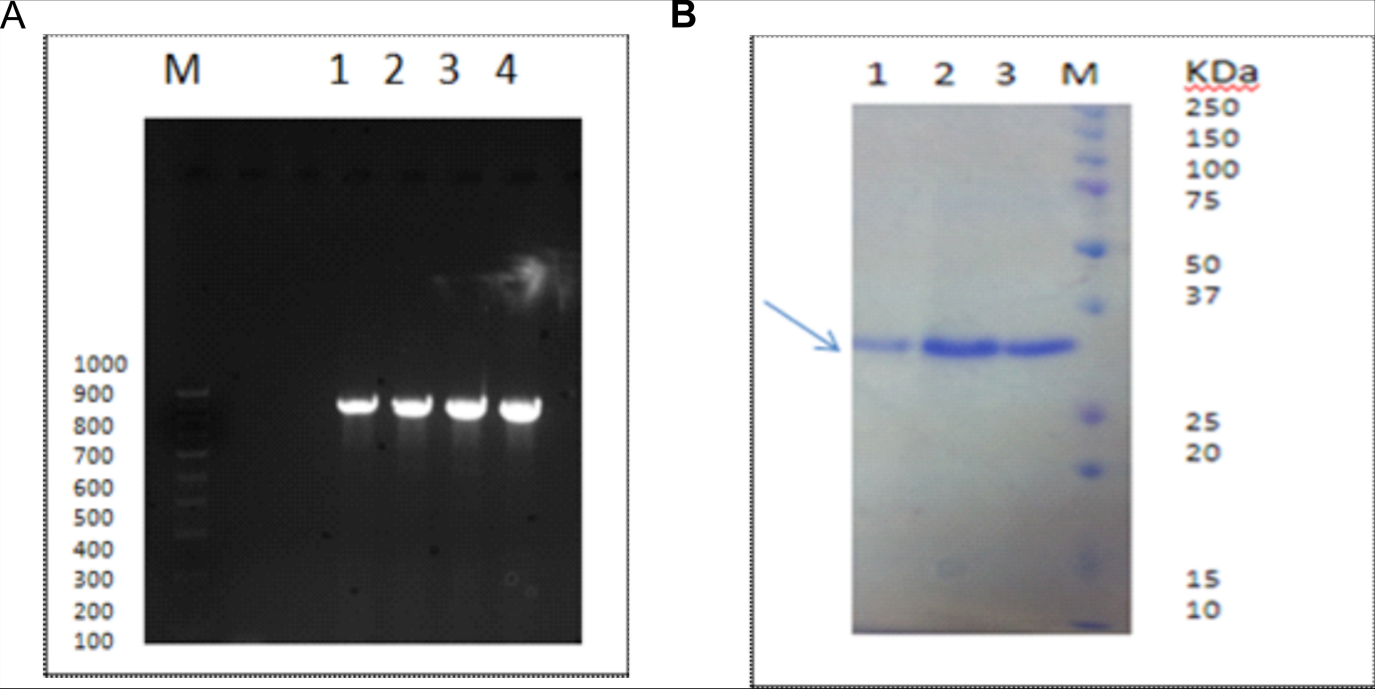
A: Amplification of gene coding for Hypothetical protein (M - 100bp marker, Lane 1 to 4- amplified gene); B: SDS PAGE showing the presence of a single band corresponding to the molecular weight (~33 KDa). Lane M- precision plus dual color marker, Lane 1 to 3- purified recombinant protein.

**Figure 9:**
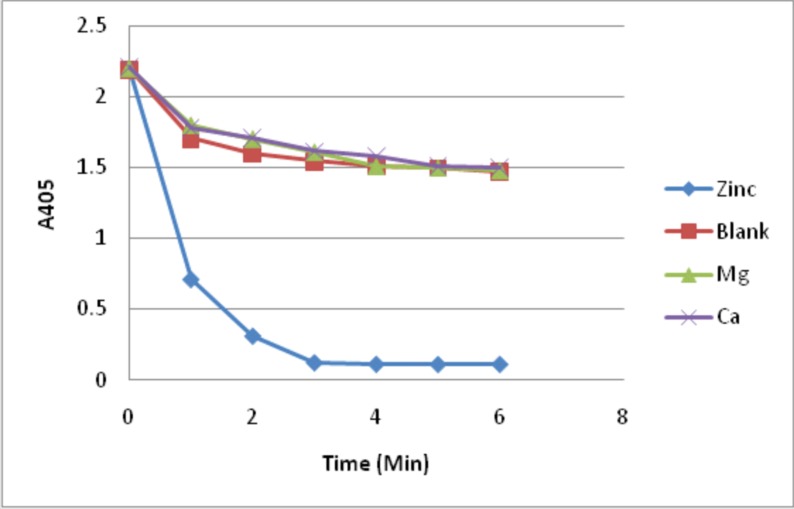
Continuous spectro-photometric rate determination for glucono-lactonase activity by measuring absorbance at 405 nm at different time intervals show change in absorbance indicating the conversion of para-nitro-phenolate to para-nitro-phenol. Activity of the recombinant protein was measured in the presence of different divalent metal ions. All data represent mean ± standard deviations (error bars) for three separate trials.

The amplified gene (shown in Figure 8A) was recovered and confirmed by gene sequencing. Upon cloning in pUC57 and subsequent subcloning in pET22b+ vector, expression was done in chemically competent E. coli BL21 cells using IPTG induction. The fusion protein was purified using nickel affinity column and further analyzed using electrophoresis on 10% SDSpolyacrylamide gel. Purified recombinant protein showed as a single band corresponding to the molecular weight (~33 KDa) as shown in figure 8B. Estimation of protein was done by Lowry's method and found to be 1.5 mg/mL. pI of protein was established by using Isoelectric focusing using a custom made apparatus, and the presence of single blue band indicated that the pI was around 4.5 (with reference to the standard). Gluconolactonase activity was determined by using D-glucono-δ- lactone as substrate by colorimetric assay. Measuring the decrease in absorbance of para nitrophenol, a pH indicator, monitored conversion of D-glucono-δ-lactone. The decrease in pH was recorded by monitoring reduction in yellow colour at 405 nm. The reaction mixture (as mentioned in materials and methods section) contained divalent metal ions zinc, magnesium and calcium in 1mM concentration separately. Figure 9 illustrates the results obtained. The specific activity of the enzyme as measured when zinc was used divalent ion was 7.21 U/mg of protein. No enzyme activity was observed when magnesium or calcium replaced zinc as the divalent metal ion in the reaction mixture. Hence we suggest that zinc is the critical divalent metal ion, which contributes to the activity of gluconolactonase.

## Conclusion

Studies on SMP30/gluconolactonase/Regucalcin family is known [22, 26]. Chen et al [27] expressed and crystallized XC5397, the first bacterial gluconolactonase from Xanthomonas campestris. The protein exhibited specificity to D-glucono-δ-lactone as substrate with a requirement for calcium as a bivalent co-factor. However, gluconolactonase from Xanthomonas campestris exhibited preference to calcium. It is interesting to note that the protein showed high preference to zinc than calcium or magnesium by this study. A considerable positive correlation between the prediction and in vitro analyses of the protein is shown. A specific activity of 7.21 U/mg of protein was observed when zinc was used as divalent metal ion and no activity was observed in the presence of calcium or magnesium as cofactor (ΔA405 in the presence of magnesium or calcium were 0 and -0.002 respectively as compared to the blank which exhibits spontaneous hydrolysis). These results are in concurrence with data shown by Tarighi et al. [7].

Compounds important for cell survival are generated from glucose via secondary metabolic pathways to produce pentose phosophates (for nucleic acid sysnthesis), D-glucouronate (for detoxification) and L-ascorbic acid or Vitamin C. δ-lactone or √- lactone is the intermediates of these secondary catabolic pathways. Lactonase inter converts linear and cyclic forms of these intermediates [27]. Therefore, characterization of YVRE as a gluconolactonse is significant in terms of establishing its role in cell survival and fitness.

Glucose biosensors use glucose oxidase (GOD) for oxidation of glucose. Ogawa et al [28] observed that shrinking kinetics of a polyelectrolyte gel used in the glucose sensor improved when gluconolactonase was co-immobilized with glucose-oxidase (GOD). It was further shown that hydrolysis of D-glucono-δ- lactone (which results from the oxidation of glucose by glucose oxidase) by gluconolactonase accelerates rapid shrinking of the gel thus improving the sensitivity of the sensor. This emphasizes the industrial application of gluconolactonase from Bacillus subtilis. Thus, the industrial application of a hypothetical protein is demonstrated using advanced genomic and prediction techiques.

## Competing Interest

Authors declare that no competing interest exists.

## Author's contribution

RSV and HGN conceived the idea. RSV and HGN designed the experimental methodology while NS and HGN designed computational workflow. RSV and NS performed the experiments and collected data. RSV, NS and HGN analysed and interpreted the data. RSV and NS prepared the manuscript and HGN critically reviewed the same. All authors have read and approved the final manuscript.

## Figures and Tables

**Table 1:** Tabulation of residues within 5Å distance from the ligand.

Sl. No	4GNA-D-Xylitol	YVRE-D-Xylitol	4GNA-D_glucono 1,5-lactone	YVRE- D_glucono 1,5-lactone
1	GLU 18	VAL 12	GLU 18	VAL 12*
2	ILE 34	ILE 13	ILE 34	ILE 13
3	ARG 101*	GLU 15*	ARG 101*	GLU 15
4	ASN 103	ARG 98*	ASN 103*	ARG 98
5	GLU 121*	ASN 100*	GLU 121*	ASN 100*
6	PRO 124	THR 115	PRO 124	THR 115
7	ALA 125	SER 116	ALA 125	SER 116
8	ASN 154*	GLU 118*	ASN 154*	GLU 118
9	ASP 204*	THR 144	ASP 204*	THR 144
10	TYR 219	ASN 146*	TYR 219	ASN 146*
11		THR 162		THR 162*
12		ASP 196		ASP 196

**Table 2:** Statistics obtained through docking.

		Score	Clash	H Bonds
D-Xylitol	Template (4GNA)	-16.1453	1.2388	6
	Model	-12.4093	1.0677	6
D-glucono-1, 5 Lactone	Template (4GNA)	-24.9553	1.6776	6
	Model	-11.0251	3.4272	5

## Abbreviations

SMP: Senescence Marker Protein
RMSD: Root mean square deviation
UDP: Uridine Diphosphate
CDD: Conserved Domain database
PDB: Protein Databank
GROMACS: GROningen MAchine for Chemical Simulations
Pfam: Protein Families database
BLAST: Basic Local Alignment Search Tool
